# TSH levels within the normal range and risk of cardiovascular and all-cause mortality among individuals with diabetes

**DOI:** 10.1186/s12933-022-01698-z

**Published:** 2022-11-23

**Authors:** Ping Zhu, Guojuan Lao, Chuping Chen, Lihui Luo, Jing Gu, Jianmin Ran

**Affiliations:** 1 Department of Endocrinology and Metabolism, Guangzhou Red Cross Hospital, Jinan University, Guangzhou, 510220 China; 2 Department of Endocrinology and Metabolism, Sun Yat-Sen Memorial Hospital, Sun Yat-Sen University, Guangzhou, 510120 China; 3 Department of Medical Statistics, School of Public Health, Sun Yat-Sen University, Guangzhou, 510089 China

**Keywords:** Thyroid-stimulating hormone, Euthyroidism, Cardiovascular mortality, All-cause mortality, Cohort analysis

## Abstract

**Background:**

Evidence regarding thyroid-stimulating hormone (TSH) levels within the normal range and mortality in adults with diabetes is scarce. This study aimed to identify the association between TSH levels and cardiovascular disease (CVD) and all-cause mortality among euthyroid patients with diabetes.

**Methods:**

This prospective cohort study included 1830 adults with diabetes from the Third National Health and Nutrition Examination Survey III. Mortality outcomes were ascertained by linkage to National Death Index records through December 31, 2019. Participants were categorized by tertiles of TSH levels (low-normal, 0.39–1.30 mIU/L; medium-normal, 1.30–2.09 mIU/L; high-normal, 2.09–4.60 mIU/L). Multivariable Cox proportional hazards models were used to explore the association between TSH levels within the normal range and overall and CVD mortality. Furthermore, restricted cubic spline analyses were used to determine the nonlinear relationship between TSH levels and mortality.

**Results:**

During a median follow-up of 17.1 years, 1324 all-cause deaths occurred, including 525 deaths from CVD. After multivariate adjustment, a U-shaped relationship was observed between TSH levels in euthyroid status and all-cause or CVD mortality among patients with diabetes (both P < 0.05 for nonlinearity). Compared with participants with medium-normal TSH levels, those with high-normal TSH levels had a significantly higher risk of all-cause (hazard ratio, 1.31; 95% confidence interval, 1.07–1.61) and CVD (1.52; 1.08–2.12) mortality. Similarly, low-normal TSH levels also increased all-cause (1.39; 1.12–1.73) and CVD (1.69; 1.17–2.44) mortality risk. In stratum-specific analyses, we found that high-normal TSH levels were associated with higher mortality risk in younger (< 60 years) patients with diabetes but not in older (≥ 60 years) participants.

**Conclusion:**

Low- and high-normal serum TSH levels were associated with increased all-cause and CVD mortality in euthyroid adults with diabetes. Further studies are needed to confirm the present observation in a wider population.

**Supplementary Information:**

The online version contains supplementary material available at 10.1186/s12933-022-01698-z.

## Background

Over the past several decades, diabetes has become a significant challenge for healthcare professionals worldwide. The estimated global prevalence of diabetes in adults was 10.5% in 2021, rising to 12.2% in 2045 [1, 2]. In addition, the incidence of cardiovascular disease (CVD) and death is two- to four-fold higher in people with diabetes than in those without diabetes [3]. Identifying novel, modifiable risk factors in patients with diabetes may be important in preventing or mitigating premature mortality.

Thyroid dysfunction is more frequent in patients with diabetes than in the general population [4, 5]. Thyroid-stimulating hormone (TSH), a hormone that primarily regulates the metabolism of the thyroid, has been associated with glycemic control and cardiovascular events partly explained by dyslipidemia, hypertension, and insulin sensitivity [4, 6, 7].

Several epidemiological studies have demonstrated an association of TSH levels within the normal range with mortality in the general population [8–13]. Some studies support unfavorable results, while others indicate no association. Recently, Kim et al. [14] analyzed the population of the National Health and Nutrition Examination Survey (NHANES) with a median follow-up of 23 years and reported that higher TSH levels in the euthyroid state increased all-cause and CVD mortality risk in individuals with nonalcoholic fatty liver disease. Another prospective study of 1265 patients with type 2 diabetes who had severe cardiovascular complications (204 deaths occurred) showed no association of TSH level in the normal range with the risk of myocardial infarction, vascular death, or all-cause mortality [15]. At baseline, 69.3% of the people in this cohort had a history of vascular disease. It is plausible that the additional influence of thyroid dysfunction is underestimated because patients with diabetes are already at a high risk of CVD events and death.

Moreover, relevant confounders such as lifestyle factors, iodine status, thyroid autoantibodies, and inflammation were not considered in the study mentioned above. To our knowledge, evidence regarding the potential burden of variation in TSH levels within the normal range on mortality risk among patients with diabetes is scarce.

Therefore, we aimed to evaluate the association between TSH levels within the normal range and all-cause and CVD mortality risks among euthyroid adults with diabetes from a large nationally representative sample.

## Methods

### Study population

Data were extracted from the NHANES database, which employs a complex, stratified, multistage, probability cluster design to assess the health and nutrition status of a nationally representative sample of the United States (US). The NHANES is widely used as a prospective cohort study by linking it to the National Death Index database [16]. Detailed information on the survey’s sampling procedure and data collection can be found elsewhere [17]. Participants were selected randomly using a complicated statistical method to conduct personal structured interviews at home, physical examinations at mobile examination facilities, and laboratory measurements [18]. The protocol for NHANES was approved by the National Center for Health Statistics and Ethics Review Board. All participants provided written informed consent.

**Fig. 1:**
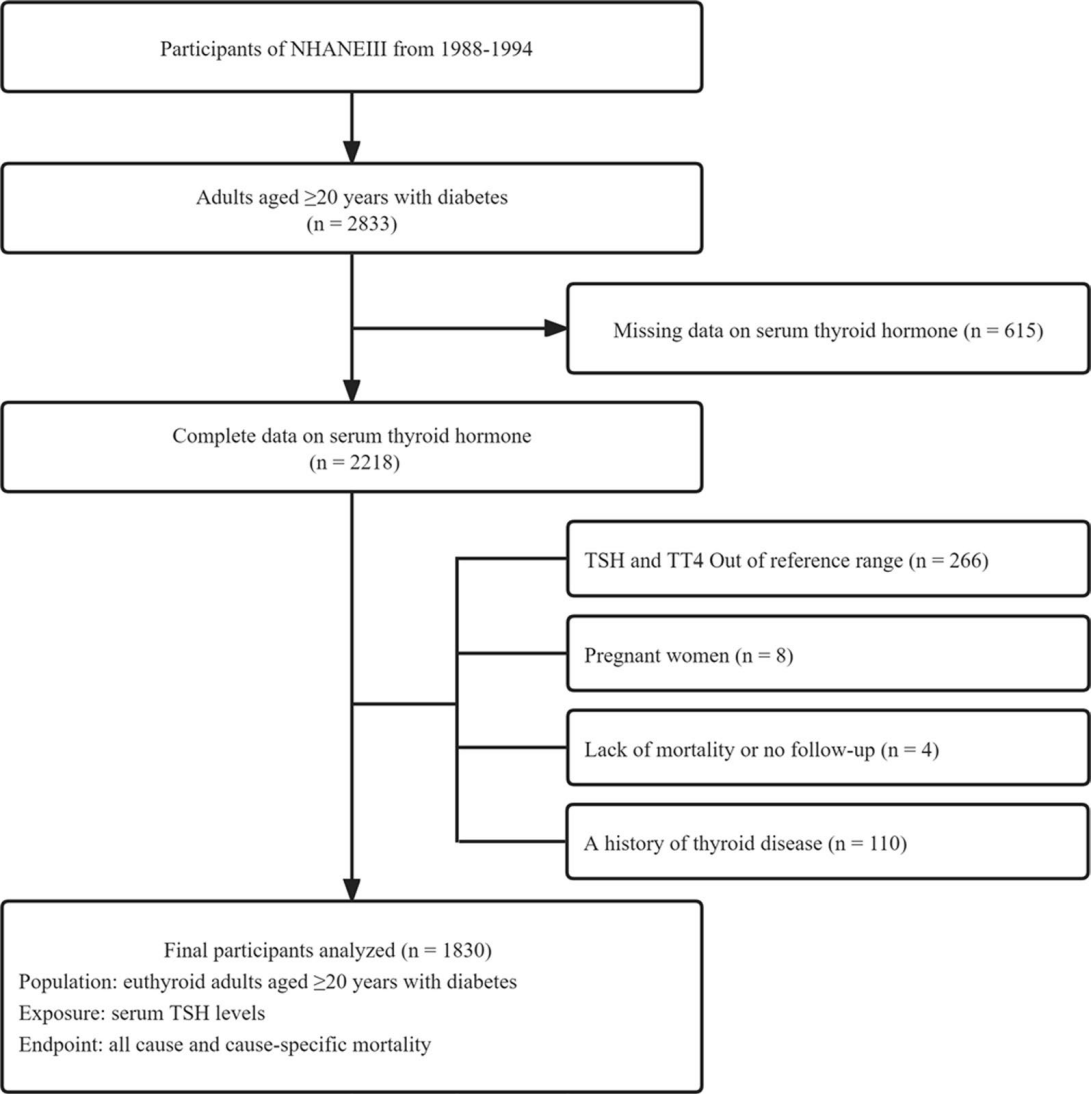
Flowchart showing the selection of eligible participants

In total, 2833 adults with diabetes aged ≥ 20 were enrolled in the NHANES III (1988–1994). The ascertainment of diabetes included a self-report of physician diagnosis, fasting plasma glucose level ≥ 7.0 mmol/L (126 mg/dL), 75-g oral glucose tolerance test result ≥ 11.1 mmol/L (200 mg/dL), or glycated hemoglobin A1c (HbA1c) level ≥ 6.5% (48 mmol/mol) [19–21]. Participants without data on serum thyroid hormone levels (n = 615), TSH and total thyroxine (TT_4_) levels outside the reference range (n = 266), those with a history of thyroid disease at baseline (n = 110), those lost to follow-up (n = 4), and those who self-reported as being pregnant (n = 8) were excluded from the study. Therefore, 1830 participants were included in the main analysis (Fig. 1).

### Definition of serum thyroid function

The serum TSH levels of participants were determined using a chemiluminescence immunoassay, and TT_4_ levels were measured using radioimmunoassay. A specific laboratory method is available on the NHANES website. The reference ranges for TSH and TT_4_ levels in the NHANES III were 0.39–4.60 mIU/L and 4.5–13.2 µg/dL, respectively. Euthyroidism was defined as serum TSH and TT_4_ levels within the reference range. Considering the possible U-shaped relationship between TSH levels and mortality [8], we categorized participants with euthyroidism into tertiles based on TSH levels: low-normal group, 0.39–1.30 mIU/L; medium-normal group, 1.30–2.09 mIU/L; and high-normal group, 2.09–4.60 mIU/L.

## Ascertainment of outcomes

The primary outcomes in our study were mortality from all causes and CVD, which were derived from the National Death Index through December 31, 2019. The follow-up period was calculated from the date of serum thyroid hormone measurement until death or the end of the follow-up, whichever occurred first. Death from CVD was identified as codes I00–09, I11, I13, I20–51, and I60–69 using the International Classification of Diseases, Tenth Revision (ICD-10).

### Assessment of covariates

Baseline data on demographics and lifestyle factors such as age, sex, race/ethnicity, education, family income, drinking status, smoking status, body mass index (BMI), Healthy Eating Index (HEI), and physical activity were acquired using established protocols [21, 22]. BMI was defined as the ratio of body weight to height squared, expressed in kg/m^2^, and was categorized as < 25, 25–30, or ≥ 30. The family income status was defined by the family income-to-poverty threshold ratio, and a low ratio indicated a higher degree of poverty. The drinking status was categorized as nondrinker or drinker. HEI scores, which represent compliance with the Dietary Guidelines for Americans, were used to evaluate diet quality. Higher scores suggested a healthier diet. Physical activity was divided into three groups based on the intensity and frequency of leisure-time physical activity (LTPA) [22]: inactive (no LTPA), insufficiently active (1–3 times a week and ≥ 6 metabolic equivalents (METs) or 1–5 times a week and 3–6 METs), and active (those who had more LTPA than those mentioned above). Frailty was defined using the previously validated five-item frailty phenotype sore first proposed by Fried et al. [23, 24]. The five items of frailty are as follows: (1) slow walking, defined as the slowest fifth of a timed 8-foot walk; (2) weakness, defined as having trouble lifting or carrying something as heavy as 10 pounds; (3) exhausting, defined as having trouble walking from one room to another on the same level; (4) low physical activity, defined as being less active when asked the question “When compared to most men/women of your age, would you say that you are less active, about the same or more active?”; (5) low body weight, defined as BMI less than 18.5 kg/m^2^. Participants were considered frail if they met three or more of the criteria listed above. The Chronic Kidney Disease Epidemiology Collaboration equation (CKD-EPI) was used to determine the estimated glomerular filtration rate (eGFR). Additionally, urinary iodine, anti-thyroid peroxidase antibody (TPO-Ab), insulin, HbA1c, total cholesterol (TC), high-density lipoprotein cholesterol (HDL-C), and C-reactive protein (CRP) levels were detected at the baseline survey [25]. The homeostasis model assessment of insulin resistance (HOMA-IR) was calculated as fasting insulin (μIU/mL) multiplied by fasting glucose (mmol/L) divided by 22.5 [26].

### Statistical analysis

To account for the multistage-sampling methodology of the NHANES, sample weights, pseudo-stratum, and primary sampling units were performed. Quantitative and categorical variables are presented as weighted means and percentages.

Weighted Kaplan–Meier curves were developed to illustrate the survival status of participants by tertiles of TSH levels. Survey-weighted Cox proportional hazards regression models were used to estimating the relationship between serum TSH levels (by tertiles) and all-cause and CVD mortality. Three models were constructed after adjusting for possible confounding factors. Model 1 was adjusted for demographic characteristics such as baseline age, sex, and race/ethnicity. Model 2 was additionally adjusted for BMI, education level, family income-poverty ratio, smoking status, drinking status, physical activity (inactive, insufficiently active, or active), and history of cancer. Model 3 was additionally modified for diabetes-related factors, including diabetes duration, use of glucose-lowering medications (none, insulin, oral hypoglycemic agent, or others), history of CVD or hypertension, HbA1c level, TC level, HDL-C level, and eGFR. The multiple imputation method was utilized to impute the missing values for some covariates [21].

Additionally, restricted cubic splines with three knots (25th, 50th, and 75th) were used to visualize the potentially nonlinear association between serum TSH levels and mortality in participants with diabetes after adjusting for all covariates mentioned above. In the presence of nonlinearity, the second tertile was used as the reference group to assess the relationship between serum TSH levels and mortality.

Stratified analyses were further applied according to several potential confounding variables, including age, sex, race/ethnicity, history of CVD, smoking status, BMI, diabetes duration, and eGFR. Interactions were examined using product terms in models adjusted for main covariates.

Additionally, sensitivity analyses were also performed. First, considering the probable interrelationship between iodine status, thyroid autoantibodies, and TSH levels [27, 28], additional adjustments for urinary iodine, TPO-Ab, and TT_4_ were performed. Second, dietary factors or frailty may have an impact on the relationship of interest, the HEI or frailty was further adjusted in the multivariate model [29]. Third, we further adjusted for the CRP level or HOMA-IR, which could be the mediators of these associations. Fourth, the association between serum TSH levels and mortality was reanalyzed according to the TSH quintiles. Fifth, deaths in the first 2 years of follow-up were excluded. Sixth, participants with a history of CVD or cancer at baseline were excluded.

All analyses were conducted using the Complex Samples Survey module of SPSS 24.0 (IBM Corporation, Armonk, NY) and R software 4.1.2 (R Foundation for Statistical Computing, Vienna, Austria). Statistical significance was defined as a two-sided P-value of < 0.05.

## Results

### Baseline characteristics

The current study enrolled 1830 adults aged ≥ 20 with diabetes (mean age, 58.0 years; 49.2% male). During a median follow-up of 17.1 years (30,808 person-years), 1324 deaths occurred, including 525 deaths from CVD and 234 from cancer. The 10th, 25th, 75th, and 90th percentiles for the follow-up time were 3.4, 8.3, 26.2, and 28.6 years, respectively. The baseline characteristics of the study participants according to the TSH tertiles are presented in Table 1. Participants in the highest tertile of TSH levels tended to be older, women, and non-Hispanic white; to have higher education levels, HEI scores, positive TPO-Ab rate, the prevalence of CVD and hypertension; and to have lower eGFRs.

**Table 1:** Baseline characteristics of patients with diabetes in NHANES III

	Total	Serum TSH levels (mIU/L)			*P* value
		Tertile 1 (0.39–1.30)	Tertile 2 (1.30–2.09)	Tertile 3 (2.09–4.60)	
Participants, n	1830	576	642	612	
Age, years	58.0 (14.2)	54.3 (15.1)	59.0 (13.8)	60.6 (13.0)	<0.001
Age ≥ 60	1047 (50.2)	286 (39.0)	359 (51.9)	402 (59.9)	<0.001
Male	859 (49.2)	273 (52.3)	311 (50.0)	275 (45.3)	0.413
Race/ethnicity					0.040
Non-Hispanic white	670 (71.6)	177 (65.7)	244 (72.1)	249 (77.1)	
Non-Hispanic black	528 (14.2)	202 (17.1)	189 (14.9)	137 (10.6)	
Hispanic Mexican	559 (6.1)	169 (6.1)	184 (6.0)	206 (6.2)	
Others	73 (8.1)	28 (11.1)	25 (7.1)	20 (6.1)	
Education level					0.571
Less than high school	702 (22.4)	215 (25.0)	233 (22.2)	254 (19.9)	
High school or equivalent	799 (50.4)	263 (49.5)	291 (51.3)	245 (50.2)	
Higher than high school	329 (27.3)	98 (25.6)	118 (26.5)	113 (29.9)	
Smoking status					0.532
Never	847 (41.4)	267 (43.1)	296 (39.0)	284 (42.2)	
Former	651 (38.6)	196 (34.5)	222 (39.9)	233 (41.4)	
Current	332 (20.0)	113 (22.4)	124 (21.1)	95 (16.5)	
BMI, kg/m^2^	29.5 (6.2)	29.3 (6.4)	29.4 (6.0)	29.8 (6.2)	0.720
Leisure-time physical activity					0.337
Inactive	530 (21.5)	171 (24.8)	174 (17.7)	185 (22.3)	
Insufficiency active	728 (42.2)	221 (41.4)	248 (43.6)	259 (41.5)	
Active	572 (36.3)	184 (33.8)	220 (38.7)	168 (36.2)	
Diabetes duration, years					0.748
<10	1391 (79.7)	446 (80.2)	482 (78.5)	463 (80.7)	
≥ 10	439 (20.3)	130 (19.8)	160 (21.5)	149 (19.3)	
HbA1c, %,					0.757
<7.0	1085 (64.9)	352 (65.1)	376 (63.3)	357 (66.6)	
≥ 7.0	745 (35.1)	224 (34.9)	266 (36.7)	255 (33.4)	
eGFR, mL/min/1.73 m^2^					0.038
<30	46 (1.7)	15 (1.7)	13 (1.8)	18 (1.7)	
30–60	542 (28.8)	142 (25.0)	189 (26.9)	211 (34.8)	
≥ 60	1242 (69.5)	419 (73.3)	440 (71.4)	383 (63.5)	
Urinary iodine, μg/L					0.146
<100	568 (34.2)	165 (33.1)	223 (39.0)	180 (30.1)	
100–187	597 (34.0)	203 (34.0)	181 (29.2)	213 (39.0)	
≥ 187	598 (31.8)	186 (32.9)	213 (31.7)	199 (30.8)	
TPO-Ab, IU/mL					<0.001
<0.5	1642 (89.9)	535 (92.6)	599 (94.5)	508 (82.2)	
≥ 0.5	188 (10.1)	41 (7.4)	43 (5.5)	104 (17.8)	
HEI score	65.1 (14.1)	63.9 (14.7)	65.0 (14.1)	66.3 (13.4)	0.274
HDL-C, mg/dL	47.0 (15.8)	47.9 (15.2)	47.1 (14.6)	46.0 (17.6)	0.565
TC, mg/dL	222.2 (49.6)	218.1 (54.8)	224.7 (46.3)	223.5 (47.1)	0.312
Medical conditions					
Hypertension	902 (47.6)	269 (43.6)	310 (48.2)	323 (50.9)	0.208
Cardiovascular disease	327 (16.1)	80 (12.4)	112 (16.1)	135 (20.0)	0.023
Cancer	173 (12.7)	51 (10.4)	59 (14.2)	63 (13.3)	0.439

All estimates accounted for sample weights and complex survey designs. Data are presented as numbers (percentages) for categorical variables and mean (standard deviation) for continuous variables. Percentages and means are adjusted for survey weights of NHANES

*BMI* body mass index, *eGFR* estimated glomerular filtration rate, *HbA1c* glycated hemoglobin A1c, *HEI* healthy eating index, *HOMA-IR* homeostasis model assessment of insulin resistance, *NHANES* National Health and Nutrition Examination Survey, *TPO-Ab* anti-thyroid peroxidase antibody

### TSH levels within normal range and mortality risks

**Fig. 2:**
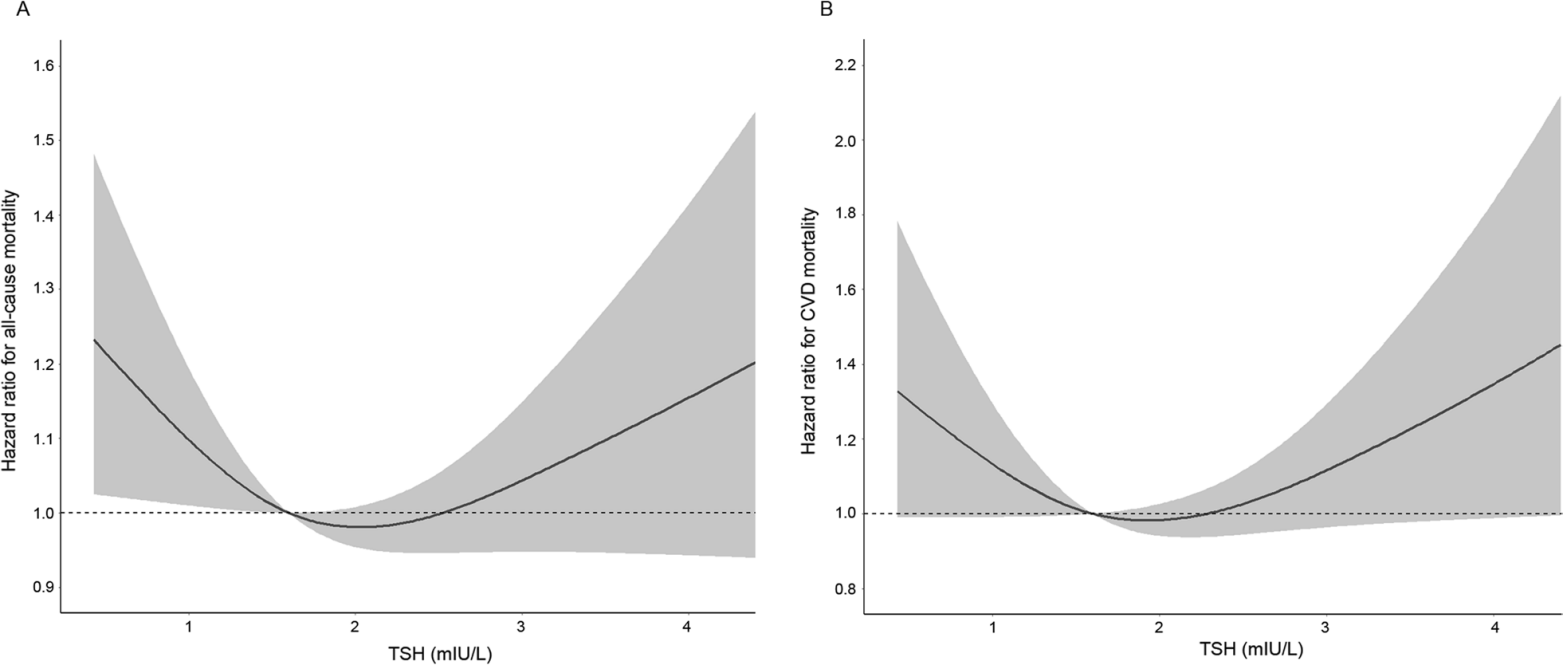
Dose–response associations between TSH levels and all-cause (A) and CVD (B) mortality risks among euthyroid patients with diabetes. Associations were investigated by multivariable Cox regression models based on restricted cubic splines. Solid lines represent hazard ratios, whereas shaded sections represent 95% CIs. Risk estimates were adjusted for age, sex, race/ethnicity, BMI, education level, family income-poverty ratio, smoking status, drinking status, physical activity, diabetes duration, glucose-lowering medication use, HbA1c, TC, HDL-C, eGFR, history of CVD, history of hypertension, and history of cancer. P-values for the nonlinear association are all < 0.05

The dose–response relationship between serum TSH levels within the normal range and all-cause and CVD mortality is shown in Fig. 2. After multivariate adjustment, we identified a U-shaped association between all-cause and CVD mortality and TSH levels (both P < 0.05 for nonlinearity).

Weighted Kaplan–Meier survival curves stratified by tertiles of TSH levels showed that patients with medium-normal TSH levels had the lowest cumulative mortality rate (Additional file 1: Figure S1). These observations were confirmed by detailed Cox proportional hazards analyses.

After full adjustment of lifestyle factors, the prevalence of CVD and hypertension, BMI, HbA1c, eGFR, TC, HDL-C, duration of diabetes, and use of glucose-lowering agents, both low- and high-normal TSH levels were significantly associated with an increased risk of all-cause and CVD mortality (Table 2). With the medium-normal TSH as the reference group, the multivariate-adjusted hazard ratio (HR) (95% confidence interval [CI]) of all-cause mortality were 1.39 (1.12–1.73) in the low-normal TSH group and 1.31 (1.07–1.61) in the high-normal group. Likewise, with the medium-normal TSH as the reference, the adjusted HR (95% CI) for CVD mortality in the low-normal and high-normal groups were 1.69 (1.17–2.44) and 1.52 (1.08–2.12), respectively. To explore the association of all-cause mortality with other cause-specific mortality, except CVD mortality, we further analyzed the relationship between TSH levels and the risk of mortality from cancer or diabetes. After multivariate adjustment, no significant association was observed between TSH levels within the normal range and cancer mortality (Additional file 2: Table S1). However, low- and high-normal TSH levels are associated with an increased diabetes mortality risk, although the results did not reach statistical significance, probably due to the limited number of events (Additional file 2: Table S1).

**Table 2:** Hazard ratio (95% CI) of all-cause and CVD mortality among patients with diabetes

	Serum TSH levels (mIU/L)		
	Low-normal (0.39–1.30)	Medium-normal (1.30–2.09)	High-normal (2.09–4.60)
All-cause mortality			
No. Deaths/total	391/576	462/642	471/612
Model 1	1.33 (1.06, 1.67)	1	1.28 (1.02, 1.61)
Model 2	1.30 (1.06, 1.61)	1	1.27 (1.04, 1.54)
Model 3	1.39 (1.12, 1.73)	1	1.31 (1.07, 1.61)
CVD mortality			
No. deaths	155	174	196
Model 1	1.57 (1.10, 2.25)	1	1.56 (1.12, 2.17)
Model 2	1.50 (1.06, 2.12)	1	1.50 (1.09, 2.07)
Model 3	1.69 (1.17, 2.44)	1	1.52 (1.08, 2.12)

**Model 1:** adjusted for age (continuous), sex (male or female), and race/ethnicity (non-Hispanic white, non-Hispanic black, Mexican American, or others);

**Model 2:** further adjusted (from Model 1) for BMI (continuous), education level (less than high school, high school or equivalent, or college or above), family income-poverty ratio (< 1, 1–3, or ≥ 3), smoking status (never, former, or current smoker), drinking status (nondrinker or drinker), physical activity (inactive, insufficiently active, or active), and history of cancer (yes or no);

**Model 3:** further adjusted (from Model 2) for diabetes duration (continuous), glucose-lowering medications usage (none, insulin, oral hypoglycemic agent, or others), history of CVD or hypertension (yes or no), HbA1c (continuous), TC (continuous), HDL-C (continuous), and eGFR (< 30, 30–60, ≥ 60 mL/min/1.73 m^2^)

### Stratified and sensitivity analyses

In the stratified analyses, a significant interaction was noted between TSH levels and baseline age with the risk of all-cause mortality among patients with diabetes (P = 0.028 for the interaction) (Table 3). For the subgroup younger than 60 years compared to the reference group, the adjusted HR (95% CI) of all-cause mortality was 1.73 (1.03–2.92) in the high-normal TSH group. However, in the subgroup aged 60 years or older compared to the reference group, the HR (95% CI) of all-cause mortality was 1.18 (0.96–1.44) in the high-normal TSH group. None of the other stratifying variables, such as sex, race/ethnicity, history of CVD, BMI, eGFR, and diabetes duration, had any effect on the association between TSH levels and all-cause mortality. We further examined the interaction between TSH levels and baseline age for the risk of CVD mortality in patients with diabetes. No significant interaction between TSH and age for the risk of CVD mortality was observed in the current study (P = 0.538 for the interaction).

**Table 3:** Stratified analyses of the associations between TSH levels and all-cause mortality among patients with diabetes

	Serum TSH levels (mIU/L)			*P* interaction
	Low-normal (0.39–1.30)	Medium-normal (1.30–2.09)	High-normal (2.09–4.60)	
Age, years				0.028
< 60	1.28 (0.78, 2.12)	1	1.73 (1.03, 2.92)	
≥ 60	1.29 (0.96, 1.73)	1	1.18 (0.96, 1.44)	
Sex				0.420
Male	1.75 (1.27–2.40)	1	1.44 (1.10, 1.89)	
Female	1.11 (0.85, 1.45)	1	1.19 (1.01, 1.45)	
Race/ethnicity				0.674
Non-Hispanic white	1.62 (1.23, 2.15)	1	1.50 (1.16, 1.94)	
Others	1.10 (0.89, 1.37)	1	0.99 (0.74, 1.32)	
Smoking status				0.154
Never	1.30 (0.93, 1.81)	1	1.33 (1.02, 1.74)	
Ever or Current	1.50 (1.14, 1.98)	1	1.34 (1.04, 1.72)	
BMI, kg/m^2^				0.683
< 30	1.53 (1.16, 2.02)	1	1.34 (1.05, 1.72)	
≥ 30	1.28 (0.95, 1.73)	1	1.28 (0.93, 1.76)	
Diabetes duration, years				0.479
< 10	1.41 (1.10, 1.80)	1	1.30 (1.04, 1.64)	
≥ 10	1.53 (0.99, 2.34)	1	1.42 (1.05, 1.92)	
eGFR, mL/min/1.73 m^2^				0.067
< 60	1.33 (0.93, 1.90)	1	1.40 (1.09, 1.81)	
≥ 60	1.47 (1.12, 1.92)	1	1.23 (0.90, 1.68)	
History of CVD				0.257
Yes	1.69 (1.06, 2.69)	1	1.49 (1.04, 2.13)	
No	1.39 (1.09, 1.78)	1	1.29 (1.02, 1.64)	

Data are presented as HR (95% CI). Adjusted for age, sex, race/ethnicity. BMI, education level, family income-poverty ratio, smoking status, drinking status, physical activity, eGFR, HbA1c, TC, HDL-C, diabetes duration, glucose-lowering medications use, history of hypertension, history of CVD, and history of cancer, except for stratifying factors

Our main findings remained robust in several sensitivity analyses. The results remained almost unchanged when excluding deaths during the first 2 years of follow-up (Additional file 2: Table S2), repeating the primary analyses by quintiles of serum TSH levels (Additional file 2: Table S3), and further excluding participants with prevalent CVD or cancer at baseline (Additional file 2: Tables S4 and Additional file 2: Table S5). Consistent results were observed when adjusted for urinary iodine concentration, TPO-Ab, or TT_4_ (Additional file 2: Table S6). The associations did not materially change when HEI scores, frailty, HOMA-IR, or CRP levels were further adjusted (Additional file 2: Tables S7 and Additional file 2: Table S8). A similar association was observed when the follow-up period was 10 years, although the results did not reach statistical significance, which could be due to reduced power (Additional file 2: Table S9).

## Discussion

In this cohort study of US adults with diabetes, with nearly two decades of follow-up, we found that low- and high-normal serum TSH levels within the normal range were associated with increased all-cause and CVD mortality risks. These findings reflect a U-shaped relationship between normal-range TSH levels and mortality, independent of established risk factors, such as lifestyle factors, diabetes duration, and glycemic control. In addition, age-stratified analyses showed a different degree of elevated all-cause mortality risk in younger patients with high-normal TSH levels. Finally, several stratified and sensitivity analyses confirmed the reliability of our findings.

Many epidemiological studies have investigated the association between serum TSH levels within the normal range and mortality in the general population with inconsistent findings [8–10, 12]. For instance, a prospective cohort study including 12,584 US euthyroid individuals from the NHANES III found that high-normal TSH levels were associated with an increased risk of all-cause and CVD mortalities compared to medium-normal TSH levels [8]. In contrast, other studies have found no associations between TSH levels within the normal range and mortality in the general population. For example, a recent population-based cohort study of South Koreans (n = 212,456) with a median follow-up of 4.3 years found that serum TSH levels within the normal range were not associated with mortality endpoints [10]. Interestingly, Cappola et al. found that higher TSH or lower FT_4_ levels within the euthyroid range were associated with a lower risk of CVD and all-cause mortality in older adults [13]. This disparity likely stems from observational studies being susceptible to confounding variables and different populations. Additionally, these studies were conducted in the general population, and the reference values for serum TSH levels differed.

However, among individuals with diabetes with a high incidence of thyroid dysfunction and an increased risk of CVD [6], evidence is scarce regarding the possible health outcomes of TSH levels within the normal range, particularly in terms of mortality. A recent cohort study from the Second Manifestations of ARTerial disease in the Netherlands explored the association between serum TSH levels within the normal range and mortality among 1265 patients with diabetes, 69.3% of whom had vascular disease [15]. The results showed no relationship between TSH levels within the reference range and vascular and all-cause mortality risk, possibly because these patients were already at a high risk of CVD, and the additional effect of thyroid dysfunction on mortality was diminished. In another hospital-based study involving 803 patients with type 2 diabetes with a 2.5-year follow-up in China [30], only 15 deaths occurred, which showed no significant association between TSH levels within the normal range and all-cause mortality. Notably, the weakness of these studies was the short duration of follow-up and inadequate adjustment of some significant confounders, such as lifestyle variables and the duration of diabetes, which may have confounded the association between TSH levels and health outcomes among patients with diabetes.

In this prospective study, we used a nationally representative US sample of euthyroid individuals with diabetes and controlled for additional potential confounding variables, such as physical activity, urinary iodine level, TPO-Ab, diabetes duration, diabetic medication use, and HOMA-IR. A U-shaped relationship was observed between serum TSH levels within the normal range and all-cause and CVD mortality. Low- and high-normal TSH levels were associated with an elevated risk of all-cause and CVD mortality among individuals with diabetes.

In the general population, the serum TSH distribution shifted progressively toward higher concentrations with aging [31]. The prevalence of subclinical hypothyroidism may be overestimated unless an age-specific TSH cut-off is implemented, which would primarily harm the elderly. It would be more reasonable to investigate the association between TSH levels and mortality using an age-specific TSH cut-off for each age category. However, a much larger sample size is needed. In this study, we used stratum-specific analysis to investigate the impact of age on results. Other studies have used similar methods to investigate the link between TSH levels and health outcomes [8, 11]. We found that high-normal TSH levels were associated with higher mortality risk in young (< 60 years) patients with diabetes but not in the elderly (≥ 60 years) participants. This may be attributed to individuals of different ages may respond differently to the hypothalamic-pituitary feedback system or adrenergic stimulation, both of which are weakened in the elderly [32, 33]. In addition, the increased all-cause mortality in young participants may be due to the unrecognized evolution to hypothyroidism in subjects with high-normal TSH levels. Nonetheless, more studies are warranted to clarify the underlying mechanisms between TSH levels and mortality across different age groups. This may have implications for future research (obtaining data on younger patient populations) and clinical practice (maybe not intervening on TSH levels within the normal range in older patients who may be less tolerant due to comorbidities).

It is worth noting that the participants studied in our study had all-cause cumulative mortality of 72.3% (1324/1830). The possible explanations for the high cumulative mortality are as follows: First, NHANES III was conducted from 1988 to 1994, and older people were oversampled to provide sufficient numbers for studies [34]. In our study, 57.2% (1047/1830, no weighted) of participants were over 60 years old, and the higher mortality rate in the elderly participants may have affected the results. Second, the participants of our study were patients with diabetes, whose all-cause mortality was 2.17 times higher than non-diabetes [35]. During the follow-up period, all-cause mortality of our study for diabetic patients was 43 deaths per 1000 person-years (1324/30,808). This result is similar to that recently reported by Visaria et al. in a US group of 13,883 diabetes patients, where the all-cause mortality was 41 deaths per 1000 person-years [36].

The possible mechanisms underlying our findings are explained below. First, the thyroid hormone plays an essential role in various physiological and pathological processes, and have a direct impact on cardiovascular risk factors by regulating myocardial contraction, endothelial function, and inflammatory pathways [37, 38]. In addition, thyroid hormone deficiency may reduce calcium cycling and ion transporter activity in cardiomyocytes, accompanied by changes in nitric oxide production, impaired endothelial function, and cardiac dysfunction [38].

The strengths of this study include its relatively large sample size, a multitude of exposures and potential confounders, and a representative sample of US adults with diabetes. However, certain limitations should be considered. First, as an observational study, our analysis should be interpreted as exploratory. No data have shown the potential clinical benefits of therapy for those individuals with TSH outside the optimal range. Second, serum thyroid hormone levels were detected only once, which may not indicate long-term exposure. Thus, the exact relationship of interest may have been underestimated. Third, the present study was conducted on a representative sample from the US, and caution should be exercised when generalizing the findings to other populations. Fourth, owing to the lack of FT_4_ levels in the NHANES III dataset, euthyroidism was determined using serum TSH and TT_4_ levels. Fifth, the dataset did not have detailed information on the time of blood sampling, although TSH secretion may have a circadian rhythm [39]. Finally, residual confounding factors could not be eliminated, although adjusting for several relevant variables.

## Conclusions

In conclusion, evidence from a nationally representative cohort revealed that low- and high-normal serum TSH levels were associated with increased all-cause and CVD mortality risk in euthyroid adults with diabetes. TSH levels and mortality have a nonlinear relationship with a U-shaped curve. Further studies are needed to confirm the present observation in wider populations with diabetes.

## Supplementary Information

**Additional file 1**: **Figure S1**. Weighted Kaplan–Meier survival curves for all-cause (A) and CVD (B) mortality based on tertiles of TSH levels among patients with diabetes. The low-normal TSH (tertile 1) indicates serum TSH levels of 0.39 to 1.30 mIU/L; medium-normal TSH (tertile 2), serum TSH levels of 1.30 to 2.09 mIU/L; and high-normal TSH (tertile 3), serum TSH levels of 2.09 to 4.60 mIU/L.

**Additional file 2**: **Table S1** Hazard ratio (95% CI) of cancer and diabetes mortality according to tertiles of serum TSH levels among patients with diabetes. **Table S2** HR (95% CI) of all-cause and CVD mortality according to serum TSH levels among patients with diabetes after excluding participants who died with 2 years of follow-up (N = 1733). **Table S3** Hazard ratio (95% CI) of all-cause and CVD mortality according to quintiles of serum TSH levels among patients with diabetes in NHANES III (N = 1830). **Table S4** Hazard ratio (95% CI) of all-cause and CVD mortality according to serum TSH levels among patients with diabetes after excluding participants with history of CVD (N = 1503). **Table S5** Hazard ratio (95% CI) of all-cause and CVD mortality according to serum TSH levels among patients with diabetes after excluding participants with history of cancer (N = 1657). **Table S6** Hazard ratio (95% CI) of all-cause mortality according to serum TSH levels among patients with diabetes with further adjustment for urinary iodine, TPO-Ab, or TT_4_. **Table S7** Hazard ratio (95% CI) of all-cause mortality according to serum TSH levels among patients with diabetes with further adjustment for HEI, HOMA-IR or CRP. **Table S8** Hazard ratio (95% CI) of all-cause and CVD mortality according to serum TSH levels among patients with diabetes with further adjustment for frailty (N = 1333). **Table S9** Hazard ratio (95% CI) of all-cause mortality according to serum TSH levels among patients with diabetes after 10-year follow-up (N = 1830).

## Data Availability

The datasets used and analyzed during the current study are available from the corresponding author upon reasonable request.

## Abbreviations

BMI: Body mass index
CKD-EPI: Chronic kidney disease epidemiology collaboration equation
CVD: Cardiovascular disease
CI: Confidence intervals
CRP: C-reactive protein
eGFR: Estimated glomerular filtration rate
HbA1c: Glycosylated hemoglobin A1c
HEI: Healthy eating index
HRs: Hazard ratios
HDL-C: High-density lipoprotein cholesterol
HOMA-IR: Homeostasis model assessment of insulin resistance
LTPA: Leisure-time physical activity
METs: Metabolic equivalents
NHANES: National health and nutrition examination survey
TC: Total cholesterol
TT_4_: Total thyroxine
TSH: Thyroid-stimulating hormone
TPO-Ab: Thyroid peroxidase antibody
