# Supplementary figures and images for: TSH levels within the normal range and risk of cardiovascular and all-cause mortality among individuals with diabetes
Source: Cardiovasc Diabetol. 2022 Nov 23;21:254. doi: 10.1186/s12933-022-01698-z (PMC9682658; doi:10.1186/s12933-022-01698-z)

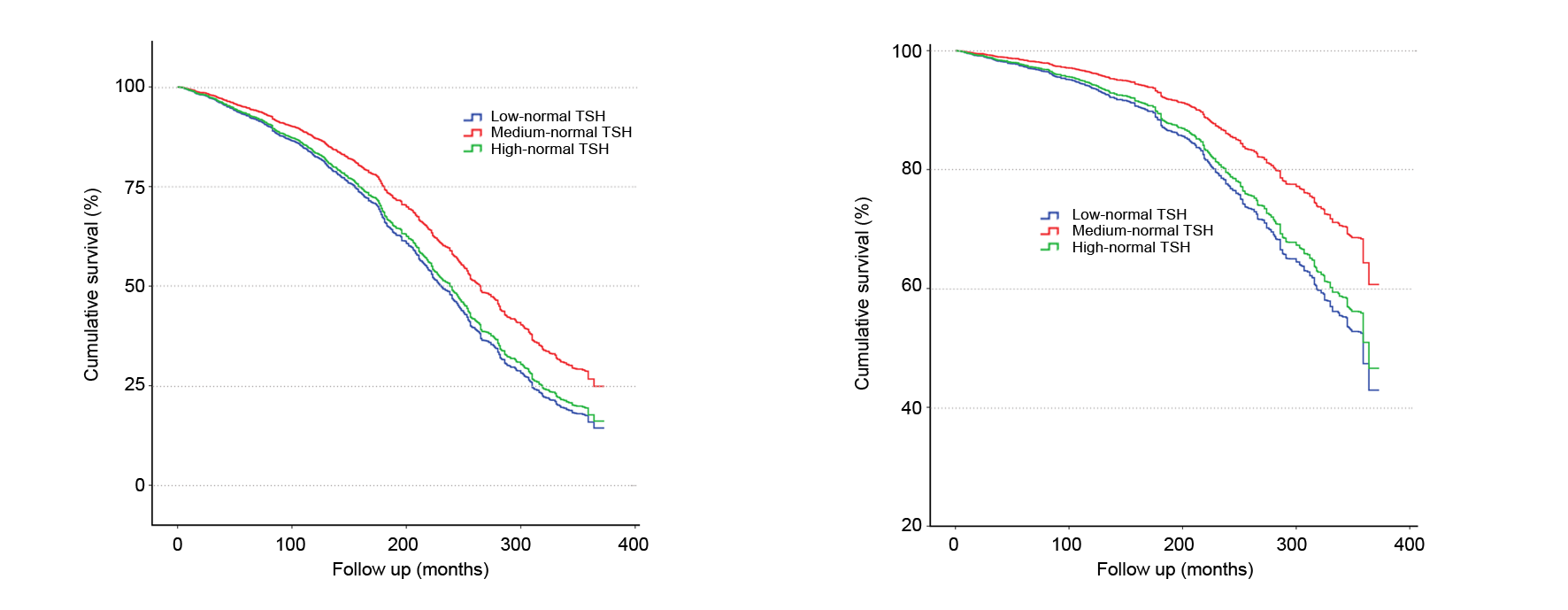

Supplement: Supplementary file 1 — Additional file 1: Figure S1. Weighted Kaplan–Meier survival curves for all-cause (A) and CVD (B) mortality based on tertiles of TSH levels among patients with diabetes. The low-normal TSH (tertile 1) indicates serum TSH levels of 0.39 to 1.30 mIU/L; medium-normal TSH (tertile 2), serum TSH levels of 1.30 to 2.09 mIU/L; and high-normal TSH (tertile 3), serum TSH levels of 2.09 to 4.60 mIU/L. [file 12933_2022_1698_MOESM1_ESM.tif]
