# Supplementary material for: TSH levels within the normal range and risk of cardiovascular and all-cause mortality among individuals with diabetes
Source: Cardiovasc Diabetol. 2022 Nov 23;21:254. doi: 10.1186/s12933-022-01698-z (PMC9682658; doi:10.1186/s12933-022-01698-z)
Supplement: Supplementary file 2 — Additional file 2: Table S1 Hazard ratio (95% CI) of cancer and diabetes mortality according to tertiles of serum TSH levels among patients with diabetes. Table S2 HR (95% CI) of all-cause and CVD mortality according to serum TSH levels among patients with diabetes after excluding participants who died with 2 years of follow-up (N = 1733). Table S3 Hazard ratio (95% CI) of all-cause and CVD mortality according to quintiles of serum TSH levels among patients with diabetes in NHANES III (N = 1830). Table S4 Hazard ratio (95% CI) of all-cause and CVD mortality according to serum TSH levels among patients with diabetes after excluding participants with history of CVD (N = 1503). Table S5 Hazard ratio (95% CI) of all-cause and CVD mortality according to serum TSH levels among patients with diabetes after excluding participants with history of cancer (N = 1657). Table S6 Hazard ratio (95% CI) of all-cause mortality according to serum TSH levels among patients with diabetes with further adjustment for urinary iodine, TPO-Ab, or TT4. Table S7 Hazard ratio (95% CI) of all-cause mortality according to serum TSH levels among patients with diabetes with further adjustment for HEI, HOMA-IR or CRP. Table S8 Hazard ratio (95% CI) of all-cause and CVD mortality according to serum TSH levels among patients with diabetes with further adjustment for frailty (N = 1333). Table S9 Hazard ratio (95% CI) of all-cause mortality according to serum TSH levels among patients with diabetes after 10-year follow-up (N = 1830). [file 12933_2022_1698_MOESM2_ESM.docx]

**Additional file 2: Table S1** Hazard ratio (95% CI) of cancer and diabetes mortality according to tertiles of serum TSH levels among patients with diabetes

|  | **Serum TSH levels (mIU/L)** | | |
| --- | --- | --- | --- |
|  | **Low-normal (0.39-1.30)** | **Medium-normal (1.30-2.09)** | **High-normal (2.09-4.60)** |
| **Cancer mortality** | | | |
| No. Deaths/total | 70/576 | 90/642 | 74/612 |
| Model 1 | 1.16 (0.77, 1.74) | 1 | 0.99 (0.57, 1.73) |
| Model 2 | 1.18 (0.78, 1.78) | 1 | 1.03 (0.63, 1.68) |
| Model 3 | 1.16 (0.76, 1.76) | 1 | 1.03 (0.64, 1.65) |
| **Diabetes mortality** | | | |
| No. deaths | 42 | 43 | 52 |
| Model 1 | 1.48 (0.81, 2.72) | 1 | 1.52 (0.72, 3.18) |
| Model 2 | 1.35 (0.72, 2.53) | 1 | 1.52 (0.77, 2.99) |
| Model 3 | 1.42 (0.75, 2.71) | 1 | 1.60 (0.88, 2.91) |

**Model 1:** adjusted for age (continuous), sex (male or female), and race/ethnicity (non-Hispanic white, non-Hispanic black, Mexican American, or others);

**Model 2:** further adjusted (from Model 1) for BMI (continuous), education level (less than high school, high school or equivalent, or college or above), family income-poverty ratio (< 1, 1–3, or ≥ 3), smoking status (never, former, or current smoker), drinking status (nondrinker or drinker), physical activity (inactive, insufficiently active, or active), and history of cancer (yes or no);

**Model 3:** further adjusted (from Model 2) for diabetes duration (continuous), glucose-lowering medications usage (none, insulin, oral hypoglycemic agent, or others), history of CVD or hypertension (yes or no), HbA1c (continuous), TC (continuous), HDL-C (continuous), and eGFR (< 30, 30–60, ≥ 60 mL/min/1.73 m^2^).

**Additional file 2: Table S2** HR (95% CI) of all-cause and CVD mortality according to serum TSH levels among patients with diabetes after excluding participants who died with two years of follow-up (N = 1,733)

|  | **Serum TSH levels (mIU/L)** | | |
| --- | --- | --- | --- |
|  | **Low-normal (0.39-1.30)** | **Medium-normal (1.30-2.09)** | **High-normal (2.09-4.60)** |
| **All-cause mortality** | | | |
| No. Deaths/total | 368/553 | 434/614 | 425/566 |
| Model 1 | 1.34 (1.06, 1.71) | 1 | 1.22 (0.96, 1.55) |
| Model 2 | 1.31 (1.04, 1.64) | 1 | 1.20 (0.98, 1.47) |
| Model 3 | 1.40 (1.11, 1.75) | 1 | 1.26 (1.02, 1.55) |
| **CVD mortality** | | | |
| No. deaths | 144 | 158 | 173 |
| Model 1 | 1.65 (1.15, 2.38) | 1 | 1.43 (1.05, 1.96) |
| Model 2 | 1.54 (1.08, 2.20) | 1 | 1.37 (1.00,1.86) |
| Model 3 | 1.73 (1.19, 2.53) | 1 | 1.38 (1.00, 1.90) |

**Model 1:** adjusted for age (continuous), sex (male or female), and race/ethnicity (non-Hispanic white, non-Hispanic black, Mexican American, or others);

**Model 2:** further adjusted (from Model 1) for BMI (continuous), education level (less than high school, high school or equivalent, or college or above), family income-poverty ratio (< 1, 1–3, or ≥ 3), smoking status (never, former, or current smoker), drinking status (non-drinker or drinker), physical activity (inactive, insufficiently active, or active), and history of cancer (yes or no);

**Model 3:** further adjusted (from Model 2) for diabetes duration (continuous), glucose-lowering medications usage (none, insulin, oral hypoglycemic agent, or others), history of CVD or hypertension (yes or no), HbA1c (continuous), TC (continuous), HDL-C (continuous), and eGFR (< 30, 30–60, ≥ 60 mL/min/1.73 m^2^).

**Additional file 2: Table S3** Hazard ratio (95% CI) of all-cause and CVD mortality according to quintiles of serum TSH levels among patients with diabetes in NHANES III (N = 1,830)

|  | **Serum TSH levels (mIU/L)** | | | | |
| --- | --- | --- | --- | --- | --- |
|  | **Quintile 1**  **(0.39-1.00)** | **Quintile 2**  **(1.00-1.40)** | **Quintile 3**  **(1.40-1.89)** | **Quintile 4**  **(1.89-2.49)** | **Quintile 5**  **(2.49-4.60)** |
| **All-cause mortality** | | | | | |
| No. Deaths/total | 223/326 | 265/387 | 261/368 | 272/360 | 303/389 |
| Model 1 | 1.40 (1.05, 1.85) | 1.25 (0.96, 1.62) | 1 | 1.15 (0.81, 1.62) | 1.22 (0.96, 1.55) |
| Model 2 | 1.38 (1.07, 1.80) | 1.15 (0.88, 1.51) | 1 | 1.09 (0.76, 1.55) | 1.24 (1.00, 1.53) |
| Model 3 | 1.46 (1.10, 1.92) | 1.21 (0.91, 1.60) | 1 | 1.18 (0.84, 1.65) | 1.28 (1.01, 1.63) |
| **CVD mortality** | | | | | |
| No. deaths | 96 | 90 | 97 | 116 | 126 |
| Model 1 | 1.62 (1.05, 2.51) | 1.25 (0.82, 1.90) | 1 | 1.17 (0.73, 1.86) | 1.60 (1.15, 2.24) |
| Model 2 | 1.64 (1.10, 2.45) | 1.09 (0.72, 1.64) | 1 | 1.12 (0.70, 1.80) | 1.55 (1.12, 2.16) |
| Model 3 | 1.83 (1.16, 2.90) | 1.12 (0.73, 1.72) | 1 | 1.19 (0.78, 1.83) | 1.56 (1.12, 2.17) |

**Model 1:** adjusted for age (continuous), sex (male or female), and race/ethnicity (non-Hispanic white, non-Hispanic black, Mexican American, or others);

**Model 2:** further adjusted (from Model 1) for BMI (continuous), education level (less than high school, high school or equivalent, or college or above), family income-poverty ratio (< 1, 1–3, or ≥ 3), smoking status (never, former, or current smoker), drinking status (nondrinker or drinker), physical activity (inactive, insufficiently active, or active), and history of cancer (yes or no);

**Model 3:** further adjusted (from Model 2) for diabetes duration (continuous), glucose-lowering medications usage (none, insulin, oral hypoglycemic agent, or others), history of CVD or hypertension (yes or no), HbA1c (continuous), TC (continuous), HDL-C (continuous), and eGFR (< 30, 30–60, ≥ 60 mL/min/1.73 m^2^).

**Additional file 2: Table S4** Hazard ratio (95% CI) of all-cause and CVD mortality according to serum TSH levels among patients with diabetes after excluding participants with history of CVD (N = 1,503)

|  | **Serum TSH levels (mIU/L)** | | |
| --- | --- | --- | --- |
|  | **Low-normal (0.39-1.30)** | **Medium-normal (1.30-2.09)** | **High-normal (2.09-4.60)** |
| **All-cause mortality** | | | |
| No. Deaths/total | 317/496 | 361/530 | 343/477 |
| Model 1 | 1.31 (0.99, 1.74) | 1 | 1.25 (0.95, 1.64) |
| Model 2 | 1.33 (1.04, 1.71) | 1 | 1.26 (1.00, 1.61) |
| Model 3 | 1.38 (1.09, 1.74) | 1 | 1.29 (1.01, 1.64) |
| **CVD mortality** | | | |
| No. deaths | 126 | 127 | 127 |
| Model 1 | 1.83 (1.21, 2.75) | 1 | 1.33 (0.93, 1.89) |
| Model 2 | 1.74 (1.20, 2.52) | 1 | 1.29 (0.89, 1.85) |
| Model 3 | 1.91 (1.29, 2.81) | 1 | 1.32 (0.90, 1.95) |

**Model 1:** adjusted for age (continuous), sex (male or female), and race/ethnicity (non-Hispanic white, non-Hispanic black, Mexican American, or others);

**Model 2:** further adjusted (from Model 1) for BMI (continuous), education level (less than high school, high school or equivalent, or college or above), family income-poverty ratio (< 1, 1–3, or ≥ 3), smoking status (never, former, or current smoker), drinking status (nondrinker or drinker), physical activity (inactive, insufficiently active, or active), and history of cancer (yes or no);

**Model 3:** further adjusted (from Model 2) for diabetes duration (continuous), glucose-lowering medications usage (none, insulin, oral hypoglycemic agent, or others), history of hypertension (yes or no), HbA1c (continuous), TC (continuous), HDL-C (continuous), and eGFR (< 30, 30–60, ≥ 60 mL/min/1.73 m^2^).

**Additional file 2: Table S5** Hazard ratio (95% CI) of all-cause and CVD mortality according to serum TSH levels among patients with diabetes after excluding participants with history of cancer (N = 1,657)

|  | **Serum TSH levels (mIU/L)** | | |
| --- | --- | --- | --- |
|  | **Low-normal (0.39-1.30)** | **Medium-normal (1.30-2.09)** | **High-normal (2.09-4.60)** |
| **All-cause mortality** | | | |
| No. Deaths/total | 346/525 | 412/583 | 412/549 |
| Model 1 | 1.37 (1.06, 1.77) | 1 | 1.32 (1.03, 1.69) |
| Model 2 | 1.31 (1.04, 1.64) | 1 | 1.28 (1.02, 1.59) |
| Model 3 | 1.32 (1.04, 1.67) | 1 | 1.39 (1.10, 1.73) |
| **CVD mortality** | | | |
| No. deaths | 142 | 153 | 169 |
| Model 1 | 1.73 (1.22, 2.45) | 1 | 1.61 (1.15, 2.25) |
| Model 2 | 1.71 (1.22, 2.41) | 1 | 1.52 (1.07, 2.17) |
| Model 3 | 1.74 (1.21, 2.50) | 1 | 1.54 (1.12, 2.13) |

**Model 1:** adjusted for age (continuous), sex (male or female), and race/ethnicity (non-Hispanic white, non-Hispanic black, Mexican American, or others);

**Model 2:** further adjusted (from Model 1) for BMI (continuous), education level (less than high school, high school or equivalent, or college or above), family income-poverty ratio (< 1, 1–3, or ≥ 3), smoking status (never, former, or current smoker), drinking status (nondrinker or drinker), and physical activity (inactive, insufficiently active, or active);

**Model 3:** further adjusted (from Model 2) for diabetes duration (continuous), glucose-lowering medications usage (none, insulin, oral hypoglycemic agent, or others), history of CVD or hypertension (yes or no), HbA1c (continuous), TC (continuous), HDL-C (continuous), and eGFR (< 30, 30–60, ≥ 60 mL/min/1.73 m^2^).

**Additional file 2: Table S6** Hazard ratio (95% CI) of all-cause mortality according to serum TSH levels among patients with diabetes with further adjustment for urinary iodine, TPO-Ab, or TT_4_

|  | **Serum TSH levels (mIU/L)** | | |
| --- | --- | --- | --- |
|  | **Low-normal (0.39-1.30)** | **Medium-normal**  **(1.30-2.09)** | **High-normal (2.09-4.60)** |
| **Participants with data of urinary iodine (N = 1,763)** | | | |
| Model 1 | 1.34 (1.06, 1.70) | 1 | 1.30 (1.03, 1.65) |
| Model 2 | 1.43 (1.15, 1.78) | 1 | 1.32 (1.07, 1.63) |
| Model 2+ urinary iodine | 1.42 (1.14,1.77) | 1 | 1.31 (1.06, 1.62) |
| **Participants with data of TPO-Ab (N = 1,830)** | | | |
| Model 1 | 1.33 (1.06, 1.67) | 1 | 1.28 (1.02, 1.61) |
| Model 2 | 1.39 (1.12, 1.73) | 1 | 1.31 (1.07, 1.61) |
| Model 2 + TPO-Ab | 1.40 (1.13, 1.73) | 1 | 1.33 (1.07, 1.64) |
| **Participants with data of TT_4_ (N = 1,830)** | | | |
| Model 1 | 1.33 (1.06, 1.67) | 1 | 1.28 (1.02, 1.61) |
| Model 2 | 1.39 (1.12, 1.73) | 1 | 1.31 (1.07, 1.61) |
| Model 2 + TT_4_ | 1.39 (1.12, 1.73) | 1 | 1.31 (1.07, 1.61) |

**Model 1:** adjusted for age (continuous), sex (male or female), and race/ethnicity (non-Hispanic white, non-Hispanic black, Mexican American, or others);

**Model 2:** further adjusted (from Model 1) for BMI (continuous), education level (less than high school, high school or equivalent, or college or above), family income-poverty ratio (< 1, 1–3, or ≥ 3), smoking status (never, former, or current smoker), drinking status (nondrinker or drinker), physical activity (inactive, insufficiently active, or active), diabetes duration (continuous), glucose-lowering medications usage (none, insulin, oral hypoglycemic agent, or others), history of CVD, hypertension, or cancer (yes or no), HbA1c (continuous), TC (continuous), HDL-C (continuous), and eGFR (< 30, 30–60, ≥ 60 mL/min/1.73 m^2^).

**Additional file 2: Table S7** Hazard ratio (95% CI) of all-cause mortality according to serum TSH levels among patients with diabetes with further adjustment for HEI, HOMA-IR or CRP

|  | **Serum TSH levels (mIU/L)** | | |
| --- | --- | --- | --- |
|  | **Low-normal (0.39-1.30)** | **Medium-normal (1.30-2.09)** | **High-normal (2.09-4.60)** |
| **Participants with data of HEI (N = 1,756)** | | | |
| Model 1 | 1.33 (1.06, 1.67) | 1 | 1.25 (1.00, 1.55) |
| Model 2 | 1.40 (1.13, 1.75) | 1 | 1.30 (1.05, 1.60) |
| Model 2 + HEI | 1.40 (1.13, 1.74) | 1 | 1.30 (1.05, 1.60) |
| **Participants with data of HOMA-IR (N = 1,819)** | | | |
| Model 1 | 1.30 (1.03, 1.65) | 1 | 1.31 (1.04, 1.64) |
| Model 2 | 1.35 (1.08, 1.69) | 1 | 1.30 (1.06, 1.58) |
| Model 2 + HOMA-IR | 1.35 (1.08, 1.69) | 1 | 1.30 (1.06, 1.59) |
| **Participants with data of CRP (N = 1,823)** | | | |
| Model 1 | 1.33 (1.06, 1.66) | 1 | 1.28 (1.02, 1.61) |
| Model 2 | 1.39 (1.12, 1.72) | 1 | 1.31 (1.06, 1.61) |
| Model 2 + CRP | 1.40 (1.13,1.73) | 1 | 1.29 (1.04, 1.59) |

**Model 1:** adjusted for age (continuous), sex (male or female), and race/ethnicity (non-Hispanic white, non-Hispanic black, Mexican American, or others);

**Model 2:** further adjusted (from Model 1) for BMI (continuous), education level (less than high school, high school or equivalent, or college or above), family income-poverty ratio (< 1, 1–3, or ≥ 3), smoking status (never, former, or current smoker), drinking status (nondrinker or drinker), physical activity (inactive, insufficiently active, or active), diabetes duration (continuous), glucose-lowering medications usage (none, insulin, oral hypoglycemic agent, or others), history of CVD, hypertension, or cancer (yes or no), HbA1c (continuous), TC (continuous), HDL-C (continuous), and eGFR (< 30, 30–60, ≥ 60 mL/min/1.73 m^2^).

**Additional file 2: Table S8** Hazard ratio (95% CI) of all-cause and CVD mortality according to serum TSH levels among patients with diabetes with further adjustment for frailty (N = 1,333)

|  | **Serum TSH levels (mIU/L)** | | |
| --- | --- | --- | --- |
|  | **Low-normal (0.39-1.30)** | **Medium-normal (1.30-2.09)** | **High-normal (2.09-4.60)** |
| **All-cause mortality** | | | |
| No. Deaths/total | 292/392 | 359/462 | 390/479 |
| Model 1 | 1.34 (1.04, 1.72) | 1 | 1.30 (1.04, 1.61) |
| Model 2 | 1.39 (1.09, 1.78) | 1 | 1.34 (1.10, 1.63) |
| Model 2 + Frailty | 1.38 (1.08, 1.77) | 1 | 1.33 (1.09, 1.62) |
| **CVD mortality** | | | |
| No. deaths | 106 | 130 | 166 |
| Model 1 | 1.33 (0.87, 2.01) | 1 | 1.56 (1.09, 2.22) |
| Model 2 | 1.39 (0.92, 2.11) | 1 | 1.56 (1.08, 2.25) |
| Model 2 + Frailty | 1.39 (0.91, 2.10) | 1 | 1.56 (1.08, 2.25) |

**Model 1:** adjusted for age (continuous), sex (male or female), and race/ethnicity (non-Hispanic white, non-Hispanic black, Mexican American, or others);

**Model 2:** further adjusted (from Model 1) for BMI (continuous), education level (less than high school, high school or equivalent, or college or above), family income-poverty ratio (< 1, 1–3, or ≥ 3), smoking status (never, former, or current smoker), drinking status (nondrinker or drinker), physical activity (inactive, insufficiently active, or active), diabetes duration (continuous), glucose-lowering medications usage (none, insulin, oral hypoglycemic agent, or others), history of CVD, hypertension, or cancer (yes or no), HbA1c (continuous), TC (continuous), HDL-C (continuous), and eGFR (< 30, 30–60, ≥ 60 mL/min/1.73 m^2^).

**Additional file 2: Table S9** Hazard ratio (95% CI) of all-cause mortality according to serum TSH levels among patients with diabetes after 10-year follow-up (N = 1,830)

|  | **Serum TSH levels (mIU/L)** | | |
| --- | --- | --- | --- |
|  | **Low-normal (0.39-1.30)** | **Medium-normal (1.30-2.09)** | **High-normal (2.09-4.60)** |
| **All-cause mortality** | | | |
| No. Deaths/total | 159/576 | 180/642 | 209/612 |
| Model 1 | 1.38 (1.00, 1.90) | 1 | 1.29 (0.95, 1.75) |
| Model 2 | 1.35 (0.99, 1.86) | 1 | 1.31 (0.93, 1.85) |
| Model 3 | 1.38 (0.97, 1.96) | 1 | 1.28 (0.93, 1.75) |

**Model 1:** adjusted for age (continuous), sex (male or female), and race/ethnicity (non-Hispanic white, non-Hispanic black, Mexican American, or others);

**Model 2:** further adjusted (from Model 1) for BMI (continuous), education level (less than high school, high school or equivalent, or college or above), family income-poverty ratio (< 1, 1–3, or ≥ 3), smoking status (never, former, or current smoker), drinking status (nondrinker or drinker), and physical activity (inactive, insufficiently active, or active);

**Model 3:** further adjusted (from Model 2) for diabetes duration (continuous), glucose-lowering medications usage (none, insulin, oral hypoglycemic agent, or others), history of CVD or hypertension (yes or no), HbA1c (continuous), TC (continuous), HDL-C (continuous), and eGFR (< 30, 30–60, ≥ 60 mL/min/1.73 m^2^).
